# α-Lipoic Acid Improves Hepatic Metabolic Dysfunctions in Acute Intermittent Porphyria: A Proof-of-Concept Study

**DOI:** 10.3390/diagnostics11091628

**Published:** 2021-09-06

**Authors:** Miriam Longo, Erika Paolini, Marica Meroni, Lorena Duca, Irene Motta, Anna Ludovica Fracanzani, Elena Di Pierro, Paola Dongiovanni

**Affiliations:** 1 UOC General Medicine and Metabolic Diseases, Fondazione IRCCS Cà Granda Ospedale Maggiore Policlinico, 20122 Milan, Italy; longo.miriam92@gmail.com (M.L.); erika.paolini@unimi.it (E.P.); maricameroni11@gmail.com (M.M.); anna.fracanzani@unimi.it (A.L.F.); 2 Department of Clinical Sciences and Community Health, Università degli Studi di Milano, 20122 Milan, Italy; irene.motta@unimi.it; 3 Department of Pharmacological and Biomolecular Sciences, Università degli Studi di Milano, 20122 Milan, Italy; 4 UOC General Medicine, Fondazione IRCCS Cà Granda Ospedale Maggiore Policlinico, 20122 Milan, Italy; lorena.duca@policlinico.mi.it (L.D.); elena.dipierro@unimi.it (E.D.P.); 5 Department of Pathophysiology and Transplantation, Università degli Studi di Milano, 20122 Milan, Italy

**Keywords:** AIP, PBGD, glucose metabolism, mitobiogenesis, α-lipoic acid

## Abstract

Background: Acute intermittent porphyria (AIP) is caused by the haploinsufficiency of porphobilinogen deaminase (PBGD) enzymatic activity. Acute attacks occur in response to fasting, and alterations in glucose metabolism, insulin resistance, and mitochondrial turnover may be involved in AIP pathophysiology. Therefore, we investigated the metabolic pathways in PBGD-silenced hepatocytes and assessed the efficacy of an insulin mimic, α-lipoic acid (α-LA), as a potential therapeutic strategy. Methods: HepG2 cells were transfected with siRNA-targeting *PBGD* (siPBGD). Cells were cultured with low glucose concentration to mimic fasting and exposed to α-LA alone or with glucose. Results: At baseline, siPBGD cells showed a lower expression of genes involved in glycolysis and mitochondrial dynamics along with reduced total ATP levels. Fasting further unbalanced glycolysis by inducing ATP shortage in siPBGD cells and activated DRP1, which mediates mitochondrial separation. Consistently, siPBGD cells in the fasted state showed the lowest protein levels of Complex IV, which belongs to the oxidative phosphorylation (OXPHOS) machinery. α-LA upregulated glycolysis and prompted ATP synthesis and triglyceride secretion, thus possibly providing energy fuels to siPBGD cells by improving glucose utilization. Finally, siPBGD exposed to α-LA plus glucose raised mitochondrial dynamics, OXPHOS activity, and energy production. Conclusions: α-LA-based therapy may ameliorate glucose metabolism and mitochondrial dysfunctions in siPBGD hepatocytes.

## 1. Introduction

Acute Intermittent Porphyria (AIP) is the most common and severe form of acute hepatic porphyria (AHP), which is a heterogeneous group of hereditary metabolic disorders characterized by defects of heme biosynthesis. Clinical manifestations include acute neurovisceral attacks (abdominal pain, nausea, constipation, vomiting) occurring in response to precipitating factors (i.e., fasting), which have in common the increased demand of hepatic heme production. Heme exerts negative feedback on the first rate-limiting enzyme of biosynthetic pathway, the 5-aminolaevulinic acid (ALA) synthase 1 (ALAS1) [1,2,3,4]. Loss of regulatory heme pool causes the upregulation of ALAS1 together with the overproduction of heme precursors as ALA and porphobilinogen (PBG), which play a key role in neurotoxicity [5,6,7].

Causative mutations of AIP affect the *Hydroxymethylbilane Synthase* (*HMBS*) gene, encoding the PBG deaminase (PBGD) enzyme, and their prevalence is relatively high in the general population (≈1/1700 subjects). Notwithstanding, the clinical penetrance hovers just around 0.5–1% of AIP carriers [8], and the genetic mutations (more than 400) identified in the *HMBS* gene are not sufficient to account for the phenotypic variability. Indeed, over 90% of AIP subjects, some of whom are highly excreters of both ALA and PBG, remain asymptomatic throughout their life, suggesting that the pathophysiology of acute events may not be directly triggered by the accumulation of the neurotoxic by-products.

Alterations of glucose metabolism and mitochondrial bioenergetics have been described in AIP experimental models, but their role in precipitating the AIP symptomatology has not been demonstrated yet. Impaired glucose tolerance and low glucose availability were observed in AIP murine models exposed to porphyrinogen drugs inducing the acute attack [9,10]. Collantes et al. revealed that AIP mice aberrantly responded to caloric restriction by stimulating gluconeogenesis and ketogenesis rather than glycogenolysis in the liver [10]. *PBGD*-deficient rodents showed NADH, FADH2, and succinyl-CoA shortage, which is the primary sources for the electron flux of the respiratory chain and heme synthesis, respectively [10,11]. Furthermore, hepatic transcriptome of AIP mice revealed that the majority of differentially expressed genes during a stress-induced attack were involved in the circadian rhythm, mitochondrial biogenesis, and oxidative phosphorylation (OXPHOS), whose regulation closely depends on *peroxisome proliferator-activated receptor gamma coactivator 1-alpha* (PPARGC1A, encoding the PGC1-α transcription factor) [12].

Intravenous hemin administration represents the first-line therapy in AIP patients to manage severe episodes, even if its chronic infusion was associated with the occurrence of debilitating attacks [13]. Recently, ALAS1-directed small interfering RNA (Givorisan) was introduced for AIP treatment in subjects with recurrent attacks, although its efficacy was paralleled by increased hepatic and renal adverse complications [14]. In sum, the current standards of care are mostly addressed to reduce hepatic ALAS1 activity, although they may present several shortcomings [15].

Foregoing studies have highlighted that AIP is featured by an impaired glucose metabolism in experimental models to the extent that carbohydrate loading has been proposed as alternative strategy in patients with mild attacks. Moreover, AIP patients showed a higher prevalence of hyperinsulinemia and insulin resistance (IR) compared to control volunteers including family members without *HMBS* mutations and porphyrin accumulation [16,17], thereby opening new perspectives for the development of novel medical care aiming to improve insulin sensitivity. Consistently, a liver-targeted insulin therapy in AIP mice promoted *ALAS1* downregulation and improved glucose metabolism [16]. Therefore, this study aimed to reproduce in vitro a condition that parallels human AIP by silencing PBGD mRNA in human HepG2 hepatoma cells and investigating metabolic alterations occurring at baseline and during a stressful factor (fasting), pointing to an in-depth characterization of glucose metabolism and mitochondrial turnover. Furthermore, in the attempt to offer a novel therapeutic option for the AIP prophylaxis, we assessed the potential efficacy of a nutraceutical supplement, the insulin mimic alpha-lipoic acid (α-LA), which has been already tested for the treatment of metabolic disorders such as type 2 diabetes, turning out to be effective and safe at improving glucose handling, insulin sensitivity, and hepatic inflammation [18,19,20].

## 2. Materials and Methods

### 2.1. RNA Interference

HepG2 human hepatoma cells, which represent the most used in vitro model to study liver metabolism and related disorders, were transiently transfected for 48 h by pooling 3 different target-specific siRNA oligo duplexes (MyBioSource, Inc., San Diego, CA, USA) of human *HMBS* gene (siPBGD) at a final concentration of 10 µM. Cyclophilin B (10 µM) was used as a scramble negative control (Horizon Discovery, Waterbeach, UK). To mimic fasting, both scramble and siPBGD cells were cultured in Dulbecco’s Modified Eagle’s Medium (DMEM, Life Technologies-ThermoFisher Scientific, Waltham, MA, USA) with low glucose concentration (1.125 mg/L) for 6 h. A cultured medium of siPBGD cells was supplemented with 0.5 mM α-LA alone (siPBGD + α-LA) or in combination with 0.33 mM glucose (α-LA + Gluc) for 24 h before inducing fasting. Treatments were freshly prepared and administered when appropriate. The potential efficacy of α-LA alone and α-LA + Gluc were compared to 0.33 mM glucose (siPBGD + Gluc) [16].

### 2.2. Evaluation of PBGD Enzymatic Activity

siPBGD and scramble cells were lysed in 0.1 M Tris-HCl buffer (pH 8.0) containing 0.2% Triton-X 100. Then, 50 µL cell lysates were incubated with 200 µL 0.1 M Tris-HCl and 25 µL of 1 mM PBG for 1 h at 37 °C in order to evaluate whether PBGD enzymes could convert PBG substrate into uroporphyrin, according to the procedure of Hsiao et al. [21]. After incubation, the enzymatic reaction was stopped through 10% trichloroacetic acid and centrifuged at 13,000 rpm for 10 min. The uroporphyrin fluorescence emission (Ex/Em = 405/655 nm) was measured by a spectrofluorometer (Shimadzu Corporation, Kyoto, Japan), and uroporphyrin concentration was determined using uroporphyrin I standard calibrator (Sigma-Aldrich, St. Louis, MO, USA). The PBGD activity (pmol Uro/h) was normalized to the total amount of proteins (mg) and expressed as percentage of residual activity for each condition.

### 2.3. Gene Expression Analysis

RNA was extracted from cell cultures using Trizol reagent (Life Technologies-ThermoFisher Scientific, Carlsbad, CA, USA). One µg of total RNA was retro-transcribed with a VILO random hexamers synthesis system (Life Technologies-ThermoFisher Scientific, Carlsbad, CA, USA). Quantitative real-time PCR (qRT-PCR) was performed by an ABI 7500 fast thermocycler, using the TaqMan Universal PCR Master Mix (Life Technologies, Carlsbad, CA, USA) and TaqMan probes (Table S1, Supplemental materials and methods). The SYBR Green chemistry (Fast SYBR Green Master Mix; Life Technologies, Carlsband, USA) was used for cDNA amplification through human primers (Table S2, Supplemental materials and methods). All reactions were delivered in triplicate. Data were normalized to *beta-actin (ACTB)* gene expression and results were expressed as arbitrary units (AU) or fold increase as indicated in bar graphs.

### 2.4. Western Blot Analysis

Total protein lysates were extracted from cell cultures, using RIPA buffer containing 1 mmol/L Na-orthovanadate, 200 mmol/L phenylmethyl sulfonyl fluoride, and 0.02 μg/μL aprotinin. Samples were pooled prior to electrophoretic separation, and all reactions were performed in duplicate. Then, equal amounts of proteins (50 μg) were separated by SDS-PAGE, transferred electrophoretically to nitrocellulose membrane (BioRad, Hercules, CA, USA), and incubated with specific antibodies overnight. At least, three independent lots of freshly extracted proteins were used for experiments. The antibodies and concentration used are listed in (Table S3, Supplemental materials and methods).

### 2.5. Statistical Analysis

Differences between two groups were calculated by two -way ANOVA, followed by post hoc *t*-test (two-tailed). Differences among multiple groups were analyzed by two-way ANOVA followed by two-stage linear step-up procedure of Benjamini, Krieger, and Yekutieli, which corrects for the number of comparisons and controls the False Discovery Rate (FDR). Adjusted (adj) *p* values < 0.05 were considered statistically significant. Statistical analyses were performed using JMP 16.0 (SAS, Cary, NC, USA) and Prism software (version 9.1, GraphPad Software).

## 3. Results

### 3.1. α-Lipoic Acid Improved Heme Production in siPBGD Cells

At baseline, *PBGD* silencing reduced both *PBGD* mRNA levels by 60% (*p* < 0.0001 at ANOVA; adj *p* < 0.0001 vs. Scramble; Figure S1A) and its enzymatic activity by 42% (*p* = 0.0001 at ANOVA; adj *p* = 0.001 vs. Scramble; Figure S1B). siPBGD cells showed lower *ALAS1* expression (*p* = 0.003 at ANOVA; *p* = 0.01 vs. Scramble; Figure S1C) and intracellular heme content compared to the scramble probably due to the delay in the heme biosynthetic pathway induced by *PBGD* downregulation (*p* < 0.0001 at ANOVA; adj *p* < 0.0001 vs. Scramble; Figure S1D).

Fasting further reduced PBGD activity (*p* = 0.0001 at ANOVA; adj *p* = 0.04 vs. siPBGD untreated (NT) and vs. fasted Scramble; Figure S1B) and promoted *ALAS1* upregulation without increasing heme content (*p* = 0.003 at ANOVA; *p* = 0.03 vs. siPBGD-NT; Figure S1C,D), as occurs in AIP subjects.

To assess the potential efficacy of α-LA as prophylactic treatment, we exposed siPBGD cells to α-LA 0.5 mM for 24 h before fasting, and its efficacy was compared to glucose supplementation (0.33 mM) at the same timing. Additionally, we explored whether α-LA could enhance glucose efficacy by evaluating the potential synergism between the two treatments.

**Figure 1:**
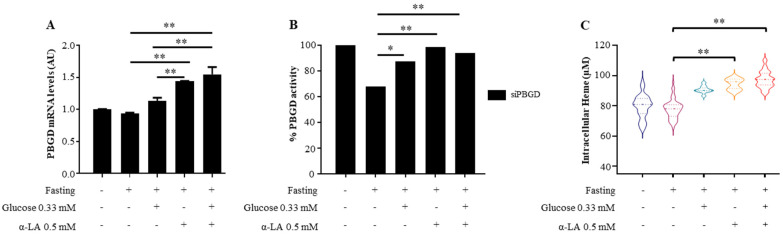
α-LA rescued heme biosynthesis in PBGD-silenced HepG2 cells. (**A**) The mRNA expression of *PBGD* was evaluated by qRT-PCR in siPBGD cells at baseline, after fasting and in the presence of glucose, α-LA, or both treatments. (**B**) The percentage of PBGD residual activity was assessed in siPBGD cells at baseline, after fasting and in the presence of glucose, α-LA, or both treatments. (**C**) Heme concentration (µM) was determined in siPBGD cells and compared to fasting, siPBGD + Gluc, siPBGD + α-LA, and to siPBGD plus α-LA + Gluc. For gene expression, data were normalized to the *ACTB* housekeeping gene and expressed as fold increase (Arbitrary Unit—AU) compared to the control group. For violin plot, data were expressed as median concentration (thick dashed lines) and interquartile range (dotted lines). At least three independent experiments were conducted. Adjusted * *p* < 0.05 and ** *p* < 0.01.

During fasting, α-LA treatment alone prevented siRNA-induced *PBGD* downregulation in terms of both mRNA levels (*p* = 0.0005 at ANOVA; adj *p* = 0.0003; Figure 1A) and protein activity *(p* = 0.03 at ANOVA; *p* = 0.005 vs. siPBGD fasted cells; Figure 1B) paralleled by increased heme availability (*p* < 0.0001 at ANOVA; adj *p* < 0.0001; Figure 1C). Moreover, α-LA showed greater ability than glucose to promote heme biosynthesis by rising PBGD levels (*p* < 0.0001 at ANOVA; adj *p* = 0.0002; Figure 1A,B) and the amount of heme (*p* < 0.0001 at ANOVA; adj *p* = 0.0004; Figure 1C).

Although α-LA + Gluc treatment ameliorated *PBGD* expression (*p* = 0.0005 at ANOVA; adj *p* = 0.0002 vs. siPBGD fasted cells; adj *p* = 0.0005 vs. siPBGD + Gluc, Figure 1A), enzymatic performance (*p* = 0.03 at ANOVA; adj *p* = 0.005 vs. siPBGD fasted cells; Figure 1B), and intracellular heme synthesis (*p* < 0.0001 at ANOVA; adj *p* < 0.0001 vs. fasted siPBGD cells + glucose; adj *p* = 0.002 vs. fasted siPBGD + α-LA; Figure 1C), the effect was quite comparable to those induced by α-LA alone, thus supporting that it may efficiently rescue heme production in hepatocytes.

### 3.2. α-Lipoic Acid Stimulates Glucose Utilization and Provides Energy Supplies during Fasting

Alterations in glucose metabolism might precipitate the AIP acute attack and compromise the efficacy of glucose therapy. However, metabolic aberrancies occurring in hepatocytes at baseline have not been described yet, and whether they could be refrained through an insulin-mimic agent has not been reported in previous studies.

In siPBGD cells, the expression of *Glucokinase* (*GCK*, *p* = 0.009 at ANOVA; adj *p* = 0.04, Figure S2A), *Phosphofructokinase* (*PFK-L*, *p* < 0.0001 at ANOVA; adj *p* < 0.0001; Figure S2B) and *Pyruvate Kinase* (*PK*, *p* < 0.0001 at ANOVA; adj *p* = 0.005, Figure S2C), genes involved in different steps of glycolysis, was lower compared to scramble. Accordingly, total ATP levels were reduced by ≈40% in siPBGD cells (*p* < 0.0001 at ANOVA; adj *p* < 0.001; Figure S2D).

Fasting further delayed glycolysis in siPBGD cells by dramatically downregulating *GCK (p* = 0.009 at ANOVA; adj *p* = 0.009, Figure S2A), *PFK-L* (*p* < 0.0001 at ANOVA; adj *p* < 0.001; Figure S2B), and *PK* expression (*p* < 0.001 at ANOVA; adj *p* < 0.001, Figure S2C) and by even inducing ATP shortfall (*p* < 0.001 at ANOVA; adj *p* < 0.001 vs. Scramble fasted cells; adj *p* = 0.0017 vs. siPBGD; Figure S2D).

**Figure 2:**
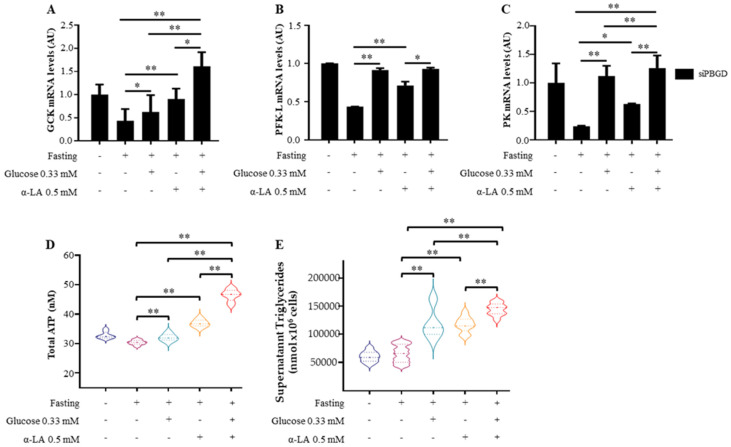
α-LA improved glucose utilization in PBGD-silenced HepG2 cells. (**A**–**C**) GCK, PFK-L and PK mRNA expression was measured in siPBGD cells at baseline, after fasting and in presence of glucose, α-LA or both treatments. (**D**) Intracellular ATP was measured in siPBGD cells and compared to fasting, siPBGD + Gluc, siPBGD + α-LA and to the combined treatment according to manufacturer ‘instruction. (**E**) Measurement of triglyceride secreted in cell supernatants of siPBGD cells with or without fasting and pre-treated with glucose, α-LA and α-LA + Gluc. For gene expression, data were normalized to *ACTB* housekeeping gene and expressed as fold increase (Arbitrary Unit-AU) compared to control group. For violin plot, data were expressed as median concentration (thick dashed lines) and interquartile range (dotted lines). At least three independent experiments were conducted. Adjusted * *p* < 0.05 and ** *p* < 0.01.

As expected, glucose administration regulated the expression of *GCK* (*p* = 0.004 at ANOVA; adj *p* = 0.02 vs. siPBGD fasted cells, Figure 2A), *PFK-L* (*p* = 0.0002 at ANOVA, adj *p* = 0.0001 vs. siPBGD fasted cells; Figure 2B), and *PK* (*p* < 0.0001 at ANOVA, adj *p* < 0.0001 vs. siPBGD fasted cells; Figure 2C), although it was ineffective at promoting ATP production, whose levels matched with those produced in siPBGD cells at baseline (*p* < 0.0001 at ANOVA; adj *p* = 0.0009 vs. siPBGD fasted cells; Figure 2D), thereby supporting that alterations of glucose metabolism occur in the presence of *PBGD* downregulation.

Interestingly, α-LA pre-treatment alone not only upregulated *GCK* (*p* = 0.004 at ANOVA; adj *p* = 0.004 vs. siPBGD fasted cells; Figure 2A), *PFK-L* (*p* < 0.0001 at ANOVA, adj *p* < 0.0001 vs. siPBGD fasted cells; Figure 2B), and *PK* mRNA levels (*p* = 0.0002 at ANOVA, adj *p* = 0.03 vs. siPBGD fasted cells; Figure 2C), but also it hugely raised the total ATP compared to either siPBGD fasted cells or to siPBGD + Gluc ones (*p* < 0.0001 at ANOVA; adj *p* < 0.0001; Figure 2D).

The combined α-LA + Gluc supplementation additively participated to promote the gene expression of glycolytic enzymes (*p* = 0.004 at ANOVA; adj *p* = 0.0001 vs. siPBGD fasted cells, adj *p* = 0.004 vs. siPBGD + Gluc, *p* = 0.02 vs. siPBGD + α-LA; *p* < 0.0001 at ANOVA, adj *p* = 0.0001 vs. siPBGD fasted cells, *p* = 0.001 vs. siPBGD + α-LA; *p* < 0.0001 at ANOVA, adj *p* < 0.0001 vs. siPBGD fasted cells, vs. siPBGD + Gluc and vs. siPBGD + α-LA; Figure 2A–C). Compared to the single treatments, α-LA + Gluc administration enriched the siPBGD cells of ATP resources (*p* < 0.0001 at ANOVA, *p* < 0.0001 adj *p* < 0.0001 vs. siPBGD fasted cells, vs. siPBGD + Gluc and vs. siPBGD + α-LA; Figure 2D) and enhanced triglyceride secretion (*p* < 0.0001 at ANOVA; *p* < 0.0001 vs. siPBGD fasted cells, vs. siPBGD + Gluc and vs. siPBGD + α-LA; Figure 2E). Therefore, it could be speculated that α-LA administration may supply *PBDG*-silenced hepatocytes of energy fuels during energy shortage by possibly improving glucose utilization.

### 3.3. α-Lipoic Acid Recovered Mitobiogenesis: The Dual Role of PGC1α

Mitochondrial dysfunctions, in terms of bioenergetic failure, have been described in AIP mice with overt symptomatology [10,11], but alterations of mitochondrial dynamics need to be further elucidated.

**Figure 3:**
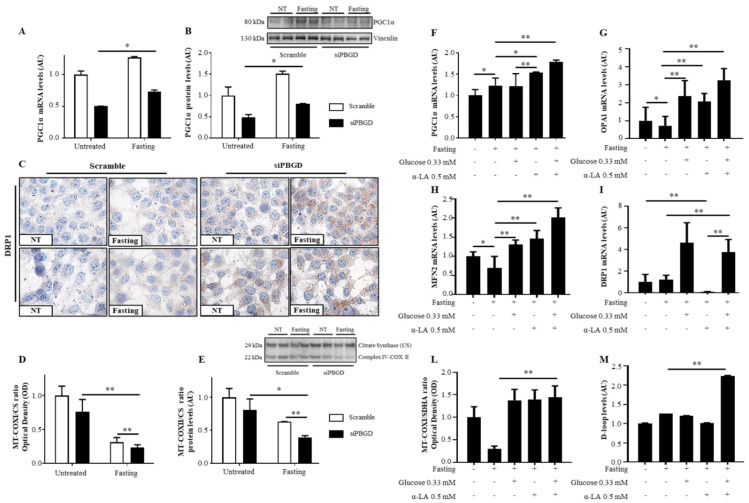
α-LA combined to glucose recovered mitochondrial dynamics in PBGD-silenced HepG2 cells. (**A**,**B**) *PGC1α* mRNA and protein levels were assessed in both scramble and siPBGD cells at baseline and after fasting by qRT-PCR and Western blot, respectively. (**C**) Cytoplasmatic localization of DRP1 protein assessed at immunocytochemistry in scramble and siPBGD cells in the absence or in presence of fasting. (**D**) Mitochondrially-encoded subunit I (MT-COXI) of Complex IV was evaluated by ELISA (λ = 600 nm) and normalized to nuclear-encoded citrate synthase levels (λ = 405 nm). (**E**) Protein levels of mitochondrially-encoded subunit II (MT-COXII) of Complex IV was assessed by Western blot and normalized to nuclear-encoded citrate synthase. (**F**–**I**) *PGC1α*, *OPA1*, *MFN2,* and *DRP1* expression was evaluated by qRT-PCR in siPBGD cells at baseline, after fasting, and in the presence of glucose, α-LA, or both treatments. (**L**) Intracellular MT-COXI of Complex IV was assessed by ELISA (λ = 600 nm) in siPBGD cells with or without fasting and pre-treated with glucose, α-LA, and α-LA + Gluc. MT-COXI protein expression was normalized to nuclear-encoded citrate synthase levels (λ = 405 nm). (**M**) D-loop levels were measured in DNA samples extracted from siPBGD cells at basal status and in fasting as well as in those treated with glucose, α-LA, and α-LA + Gluc. For gene expression, data were normalized to the *ACTB* housekeeping gene and expressed as fold increase (Arbitrary Unit—AU) compared to the control group. For Western blot, data were normalized on vinculin or citrate synthase housekeeping genes. At least three independent experiments were conducted. Adjusted * *p* < 0.05 and ** *p* < 0.01.

The mRNA and protein levels of PCG1α, master regulator of mitobiogenesis, were induced by fasting in both scramble and siPBGD cells (*p* < 0.0001 at ANOVA, adj *p* = 0.008 vs. siPBGD-NT, Figure 3A,B) accompanied by the downregulation of *Optical Atrophy 1* (*OPA1*), which mediates the fusion of mitochondrial inner membranes, and *Mitofusin 2* (*MFN2*), which joins mitochondrial outer membranes (*p* = 0.01 at ANOVA, adj *p* < 0.05 vs. Scramble-NT and vs. siPBGD-NT, Figure S3A,B). Conversely, we found that dynamin 1-like protein (DRP1), which regulates mitochondrial separation, was markedly localized in the cytoplasm of siPBGD cells in fasting condition (Figure 3C). Since DRP1 activation is usually associated to low OXPHOS capacity and energy shortfall, we assessed the expression of Complex IV of the respiratory chain, which is encoded by mtDNA and represents the core of OXPHOS functionality. In keeping with previous findings, siPBGD cells showed the lowest levels of COXI and COXII, which are both subunits composing the Complex IV (*p* < 0.0001 at ANOVA, adj *p* < 0.0001 **vs.** siPBGD-NT and *p* = 0.0007 vs. scramble fasted cells, Figure 3D; adj *p* < 0.05 vs. siPBGD-NT and *p* < 0.01 vs. scramble fasted cells, Figure 3E), thereby supporting that fasting may activate mitochondrial biogenesis and exacerbate mitochondrial injury in siPBGD cells by shifting toward fission rather than fusion.

In siPBGD cells, pre-treatment with α-LA before fasting enhanced mitochondrial biogenesis by upregulating *PCG1α*) more than glucose administration alone (*p* < 0.0001 at ANOVA, adj *p* = 0.01 vs. siPBGD fasted cells and *p* = 0.004 vs. siPBGD + Gluc, Figure 3F). The *OPA1* and *MFN2* mRNA expression were increased with the α-LA and glucose supplementation compared to siPBGD in fasting condition, showing similar levels among the two treatments (*p* < 0.0001 at ANOVA, adj *p* < 0.0001; Figure 3G,H). Unexpectedly, DRP1 expression was strongly pulled down by α-LA treatment (*p* < 0.0001 at ANOVA, adj *p* = 0.0002 vs. siPBGD-NT, *p* = 0.0001 vs. siPBGD fasted cells and *p* < 0.0001 vs. siPBGD + Gluc, Figure 3I), supporting that it may counteract the effect of fasting by switching mitochondrial biogenesis toward fusion rather than fission.

Co-treatment with α-LA + Gluc during fasting had the highest impact of *PCG1α* upregulation (*p* < 0.0001 at ANOVA, adj *p* < 0.0001 vs. siPBGD fasted cells and vs. siPBGD + Gluc, *p* = 0.009 vs. siPBGD + α-LA, Figure 3F) along with the overexpression of *OPA1* (*p* < 0.0001 at ANOVA, adj *p* < 0.0001 vs. siPBGD fasted cells and vs. single treatments, Figure 3G), *MFN2* (*p* < 0.0001 at ANOVA, adj *p* < 0.0001 vs. siPBGD fasted cells, *p* = 0.001 vs. siPBGD + Gluc and *p* = 0.0003 vs. siPBGD+ α-LA, Figure 3H), and *DRP1* (*p* < 0.0001 at ANOVA, adj *p* = 0.0003 vs. siPBGD fasted cells, Figure 3I). No differences among glucose, α-LA, and α-LA + Gluc were found at improving Complex IV abundance during fasting (*p* < 0.0001 at ANOVA, adj *p* < 0.0001 vs. siPBGD fasted cells, Figure 3L). Nonetheless, the expression of *D-loop*, which reflects mitochondrial mass, was around 2.5-fold higher only after α-LA + Gluc treatment compared to siPBGD cells with or without single treatments (*p* < 0.0001 at ANOVA, adj *p* < 0.0001 vs. siPBGD fasted cells and vs. siPBGD + Gluc and vs. siPBGD + α-LA, Figure 3M). These findings may support that α-LA + Gluc combination may additively rescue the overall mitochondrial dynamics in terms of mitochondrial mass, energy production, and mitochondrial turnover.

## 4. Discussion

The binomial association between porphyrins and acute neuropsychiatric attacks has been at the basis of AIP pathogenesis for decades. Still, there are several reports that cast doubt about the exclusive pathogenic role of heme precursors in disease, as AIP patients who accumulate ALA and PBG may not manifest any acute attack throughout their life [16,22]. Thus, other factors may be involved in the pathophysiology of the acute event. A link may exist between metabolic homeostasis and the biosynthesis of heme, as the latter is an essential precursor for the correct nutrients’ handling to meet energy requirements during basal and stressful situations.

Attempting to shed light on this issue, we silenced the *PBGD* gene in HepG2 cells (siPBGD) and characterized metabolic alterations occurring at baseline and after glucose deprivation, which is a condition that could mimic fasting in vitro. Transient *PBGD* silencing in HepG2 cells reduced *PBGD* and *ALAS1* levels paralleled by the low hepatocellular heme content, thus supporting it may reproduce the slowdown of the hepatic heme biosynthesis observed in carriers of the *HMBS* genetic mutations [23]. We revealed that siPBGD cells at baseline showed a low expression of glycolytic enzymes and mitochondrial ATP production, possibly indicating that *PBGD* downregulation may cause early aberrancies of glucose metabolism and OXPHOS functionality. The basal characterization of hepatic metabolism in AIP context has been poorly explored, and just one study reported that the hepatic transcriptomic profile was similar between AIP and the congenic wild-type (WT) mice in the absence of the stress-induced attack [12]. Nonetheless, the characterization of nutritional status of AIP subjects highlighted that the inadequate glucose and carbohydrates consumption of less than 45–60% of the total energy intake was associated with disease severity [24,25]. In addition, sub-clinical OXPHOS defects, resulting in high circulating lactate levels, were found outside of the crisis in AIP patients in clinical remission [26]. Therefore, there may be some discrepancies in gene expression at baseline between transcriptomic findings by Chen et al. [12] and ours, which was mainly due to the limitations of transient PBGD silencing in vitro, which could not fully resemble the features of a stable knockout model. However, the downregulation of glycolysis and the biochemical evaluation of energetic status of siPBGD cells could, in broad terms, mirror the energy failure featuring AIP patients at higher risk of developing symptoms [12,24,25,26]. Previous evidence has outlined that most of the dysregulated pathways in the liver that may precipitate in the AIP acute attacks are under the transcriptional regulation of PCG1α, a powerful nutrient sensor activated in response to stressful factors [27]. In the AIP context, it attempted to re-establish glucose homeostasis by activating hepatic gluconeogenesis and increasing mitochondrial mass, but it also induced ALAS1 expression [12,13,17], thereby exacerbating the porphyrins’ overproduction. In keeping with these findings, we found that the induction of fasting through glucose deprivation halved PBGD enzymatic activity at 50% and sensitized siPBGD cells to upregulate ALAS1, likely via PGC1α, without increasing heme production, similar to what has been reported in AIP rodents and patients exposed to precipitating factors [10,16].

Fasting even reduced glycolysis and total ATP levels, possibly aggravating energetic imbalance in siPBGD cells, as occurs in AIP mice during an attack that lowers glucose availability and runs into cataplerosis of the Krebs cycle, resulting in an inability to provide reducing equivalents (NADH, FADH2) to the OXPHOS [28]. Beyond bioenergetic failure, alterations of mitochondrial dynamics, including a cycle of fission and fusion events, could exacerbate metabolic dysfunctions as it regulates the intracellular mitochondrial mass, shape, and metabolic status of these organelles [29]. Here, we firstly revealed that fasting-induced PCG1α activation in siPBGD cells was accompanied by the low expression of fusion genes and overexpression of DRP1, which is the main inducer of mitochondrial separation. The fission of mitochondria is commonly associated with low OXPHOS performance and ATP synthesis consistent with the reduced expression of complex IV, which has been observed in siPBGD fasted cells. Homedan et al. showed that hepatic complexes I, II, and III, but not complex IV, decreased their activities in *PBGD*-deficient mice treated with phenobarbital, which is a drug that induces the acute episodes [11,28]. Conversely, the morphological alterations of mitochondria presenting paracristallin inclusions have been described in the liver biopsies of AIP patients [29], and a lack of complex IV activity alongside the collapse of ATP was found in the hippocampus of AIP mice knocked-in for the *Hmbs* c.500G  >  A (p.R167Q) mutation, resulting in a severe phenotype with neuropsychiatric behavior [30]. Most recently, it has been demonstrated that the deficiency of *ferrochelatase* (*FETCH*), a gene involved in the last step of heme production and responsible for the development of Erythropoietic protoporphyria, damaged both glycolysis and OXPHOS along with a decrease in mitochondrial fusion [31]. Still, glucose metabolism and mitochondrial dysfunction might precipitate the acute attacks, but not all studies share the same alterations, possibly reflecting the huge phenotypic variability of AIP symptomatology [11,28,29,30,31]. Overall, our model may support the hypothesis that fasting on one hand worsens glycolytic alterations and on the other unmasks mitochondrial damage by shifting mitochondrial dynamics toward fission rather than fusion and, consequently, contributing to OXPHOS aberrancies.

Life-threating attacks featuring AIP patients represent a crippling issue for subjects who suffer from this rare disorder and mostly for those who are predisposed to recurrent crisis. The ongoing therapies aim to modulate ALAS1 activity and even showed several constraints [14,15,17,32]. AIP patients with mild attacks may be treated with glucose solution, but there are no clear clinical data showing beneficial effects in AIP prophylaxis [16,33], which is possibly due to an impaired glucose metabolism [9,10,11]. Conversely, hemin directly represses ALAS1 activity, but its long-term exposure has been associated with hepatic iron accumulation, oxidative stress, inflammation, and liver fibrosis in AIP patients alongside refractoriness to the attacks [13,32], growing the need of novel medications. In the recent years, it has emerged that AIP patients showed hyperinsulinemia, which is a condition that appeared protective against a stress-induced attack [16]. It has been also postulated that reduced insulin levels and C-peptide were associated with AIP disease activity, which was possibly due to the essential role of insulin for the ALAS1 inhibition and blockage of porphyrin overproduction [34].

Therefore, we assessed whether treatment with an insulin sensitizer, the α-LA, alone or in combination with glucose may improve metabolic abnormalities when supplemented before fasting-induced stress with the goal to offer a potential preventive approach of acute events.

**Figure 4:**
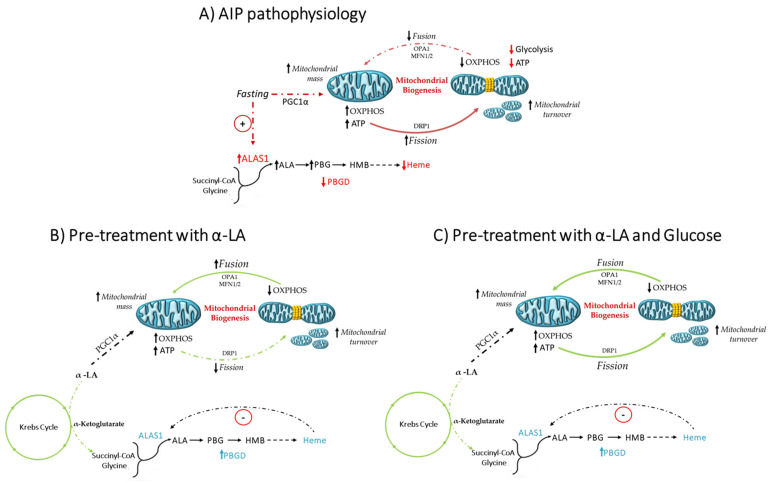
Schematic representation of metabolic alterations and the efficacy of α-LA in siPBGD cells during fasting. (**A**) Fasting induced mitochondrial biogenesis through PGC1α, possibly worsening mitochondrial injury by shunting mitochondrial dynamics toward fission, which was mediated by DRP1, and inhibiting OPA1 and MFN2, which provide the fusion of mitochondrial inner and outer membranes. The accumulation of divided mitochondria lowered OXPHOS ability and exacerbated ATP shortfall. (**B**) Pre-treatment with α-LA in siPBGD cells even promoted PGC1α activity, but it improved OPA1 and MFN2 expression, thereby enhancing mitochondrial fusion, which is a condition that could support OXPHOS activity during energy demand. (**C**) Pre-treatment with α-LA and glucose may recover the overall mitochondrial wellness by acting at multiple levels. On one hand, α-LA may improve heme biosynthesis, which is a mechanism occurring in the mitochondrial matrix. On the other hand, α-LA may enhance glycolysis, ATP production, and OXPHOS, possibly sustaining the Krebs cycle. The restoration of hepatocellular homeostasis could allow recovering mitochondrial dynamics, in terms of fusion, fission, and overall mitochondrial mass.

α-LA acts as co-factor of the α-ketoglutarate dehydrogenase in the Krebs cycle, which bridges the glycolytic–mitochondrial respiration network and provides succinyl-CoA substrate for heme biosynthesis [35,36]. The main findings of this project were that pre-treatment with α-LA, likely boosting the mitochondrial Krebs cycle, ameliorated heme biosynthesis in siPBGD cells and their energetic status by promoting ATP and lipid production. Moreover, we revealed that α-LA may counterbalance the harmful effects of fasting through the stimulation of mitochondrial elongation and inhibition of mitochondrial fission attempting to meet energy demand. Glucose administration alone even recovered the expression of glycolytic enzymes and of genes involved in mitochondrial dynamics, but it failed at improving ATP content, possibly explaining the reduced efficacy of glucose therapy reported in humans [16]. When α-LA was combined to glucose, we found that total ATP, triglyceride levels, and mitochondrial mass were increased in siPBGD cells, supporting that α-LA may additively enhance glucose efficacy and rescue the overall mitochondrial dynamics (Figure 4). By looking at translational perspectives, α-LA supplementation may potentially sustain the hepatic absorption and maintenance of glucose homeostasis from dietary carbohydrates [16].

The work showed some limitations. Firstly, the study was carried out in an in vitro model in which PBGD was transiently silenced, thereby not allowing us to perform a long-term evaluation of the metabolic profile and the efficacy of α-LA supplementation. Therefore, the siPBGD model may not fully resemble features of in vivo models and AIP patients. Secondly, the results should be confirmed in other hepatocellular cell lines, including primary hepatocytes, which could overcome the metabolic alterations characterizing the hepatoma cells as HepG2.

To conclude, emerging studies have highlighted that heme depletion may deeply affect glucose metabolism and mitochondrial dynamics [31]. The present study attempted to dissect the role of metabolic aberrancies occurring in hepatocytes in the context of *PBGD* downregulation, mimicking the AIP condition in vitro. Although α-LA was previously evaluated in the Porphyria Cutanea Tarda in hexachlorobenzene-induced porphyria as an antioxidant agent [37,38], we proposed, for the first time, its use for the prevention of AIP crisis through the improvement of hepatocellular bioenergetics. The α-LA was attractive for the management of metabolic disorders, as it did not induce significant side effects, although its efficacy and safety in AIP patients might be confirmed in clinical trials. As proof-of-concept, our findings highlighted that α-LA could re-establish glucose employment, thus restoring the cross-talk among cytosolic glycolysis and mitochondrial respiration and, therefore, the hepatocellular homeostasis.

## Data Availability

Not applicable.

## Supplementary Materials

The following are available online at https://www.mdpi.com/article/10.3390/diagnostics11091628/s1, Table S1: List of TaqMan Probes used in quantitative real-time PCR experiments; Table S2: Sequence of primers used in quantitative real-time PCR experiments; Table S3: List of antibodies and relative dilutions used in Western blotting and ICC experiments. Figure S1: PBGD downregulation reduced heme biosynthesis in HepG2 cells; Figure S2: Fasting dampened glycolysis and ATP production in siPBGD cells; Figure S3: Fasting induced downregulation of genes involved in fusion of mitochondrial membranes.
