# Supplementary material for: α-Lipoic Acid Improves Hepatic Metabolic Dysfunctions in Acute Intermittent Porphyria: A Proof-of-Concept Study
Source: Diagnostics (Basel). 2021 Sep 6;11(9):1628. doi: 10.3390/diagnostics11091628 (PMC8468570; doi:10.3390/diagnostics11091628)
Supplement: Supplementary file 1 [file diagnostics-11-01628-s001.zip › diagnostics-1347564-supplementary.pdf]

α-Lipoic Acid Improves Hepatic Metabolic Dysfunctions in Acute Intermittent Porphyria: A Proof-of-Concept Study

Miriam Longo[1,2]; Erika Paolini[1,3]; Marica Meroni[1]; Lorena Duca[4], Irene Motta[1,2]; Anna L. Fracanzani[1,5]; Elena Di Pierro[4,†]; Paola Dongiovanni[1,†]

[1]General Medicine and Metabolic Diseases; Fondazione IRCCS Cà Granda Ospedale Maggiore Policlinico, Milan, Italy.
[2] Department of Clinical Sciences and Community Health, Università degli Studi di Milano, Milano, Italy.
[3]Department of Pharmacological and Biomolecular Sciences, Università degli Studi di Milano, Milan, Italy.
[4]Fondazione IRCCS Cà Granda Ospedale Maggiore Policlinico, U.O.C Medicina Generale, Milan, Italy.
[5]Department of Pathophysiology and Transplantation, Università degli Studi di Milano, Italy.
†Equal contributors

Corresponding Author

Paola Dongiovanni
General Medicine and Metabolic Diseases, Fondazione IRCCS Cà Granda Ospedale Maggiore Policlinico, Milan, Italy,
Via Francesco Sforza 35, 20122 Milano.
Phone: +39-02-55033467. Fax +39-02-50320296.
e-mail: paola.dongiovanni@policlinico.mi.it

Supplemental Materials and Methods

Chemicals

Bovine serum albumin (BSA) was purchased from Sigma-Aldrich (St Louis, MO). Dulbecco's modified Eagle's medium (DMEM), fetal bovine serum (FBS), phosphate-buffered saline (PBS), L-Glutamine, penicillin/streptomycin, Trypsin/EDTA, Hank's balanced salt solution (HBSS), fast SYBR green master mix and lipofectamine RNAiMAX transfection reagent were obtained Life Technologies-ThermoFisher Scientific (Waltham, United States). Anti-DRP1, anti-mtCOXII, anti-Citrate Synthase and anti-vinculin primary antibodies were acquired from Abcam (Cambridge, UK). Anti-PCG1α primary antibody was obtained from Novus Biologicals (Littleton, U.S.A.). Anti-mouse IgG HRP-linked and anti-rabbit IgG HRP-linked secondary antibodies were purchased from Cell Signalling (Boston, United States). VectaMount AQ Mounting Medium was obtained from Maravai Life Sciences Inc. (United Kingdom). ATP Assay Kit (colorimetric/fluorometric), Triglycerides Quantification Assay Kit (colorimetric/fluorometric), Heme Assay Kit and MitoBiogenesis™ In-Cell ELISA Kit were bought from Abcam (Cambridge, UK). TaqMan Fast Universal PCR Master Mix No Amperase, TaqMan Copy Number Assay and Reference Assay were obtained from Applied Biosystems (United Kingdom). QIAmp DNA Mini Kit was purchased from Qiagen (Manchester, UK). α-Lipoic Acid and Glucose powders were purchased from Sigma-Aldrich (St Louis, MO).

Immunocytochemistry (ICC)

1x10^6 cells were seeded on coverslip lodged in a 6-well plate in duplicate and kept overnight in DMEM medium containing 4500 mg/L glucose, 10% Fetal Bovine Serum (FBS), 1% L-glutamine and 1% Penicillin/Streptomycin. Next, hepatocytes were fixed in 4% formalin for 15 minutes and permeabilized in 0.3% Triton-X 100. Cells were incubated in 5% Bovine Serum Albumin (BSA) for 30 minutes and with anti-DRP1 primary antibody overnight at 4°C. Then, each sample was incubated with anti-mouse HRP-conjugated secondary antibody and 3,3′-Diaminobenzidine (DAB) was provided as chromogen (brown deposits). Nucleus were counterstained with hematoxylin. Finally, samples were mounted with a drop aqueous VectaMount AQ Mounting Medium (Maravai Life Sciences Inc. United Kingdom).

Evaluation of mitochondrially DNA (mtDNA)-encoded COX-I

Mitobiogenesis In-Cell ELISA Kit Abcam (Cambridge, UK) exploits a quantitative immunocytochemistry (ICC) to measure subunit I of mtDNA-encoded Complex IV (mt-COX-I) and nuclear DNA (nDNA)-encoded succinate dehydrogenase complex flavoprotein subunit A (SDHA, otherwise known as Complex II or Citrate Synthase (CS)) protein levels in live cells and it was performed following the manufacturer' instructions [22]. Briefly, cells are seeded in a 96-well plate ($3x10^5$/well) in triplicate, fixed with 4% paraformaldehyde and permeabilized through 1X Triton-X 100. Targets of interest were detected with highly specific, well-characterized cocktail of monoclonal antibodies, which were incubated overnight at 4°C. Then, AP-labelled and HRP-labeled secondary antibodies were used to generate a colorimetric reaction that could be measured at 405 and 600 nm, respectively.

Measurement of mitochondrial ATP production

$1x10^6$ HepG2 cells were seeded in 6-well plates. Pellets were collected and homogenized in 5% IGEPAL® CA-630 (Sigma Aldrich, USA). To avoid enzymes interfering with the assay, samples were deproteinized by adding ice cold 1M perchloric acid (PCA). To neutralize PCA-induced acidification and to restore pH range at 6.5-8, 35% of potassium hydroxide (KOH) 2M was added to each sample. The excess of PCA/KOH was precipitated by centrifugating e at 13,000 g for 15 minutes at 4°C. Supernatant were collected and loaded in black walled, clear bottom plates for fluorometric detection (Ex/Em = 535/587 nm).

Quantification of mtDNA content

Genomic and mtDNA were extracted from cell lysates through QIAmp DNA Mini Kit (Manchester, UK). $1x10^6$ cells were resuspended in a Protease solution and incubated 56°C for 10 minutes to disrupt protein-DNA interactions. Total DNA was trapped onto the QIAamp silica membrane, while contaminants were removed in the flowthrough. Subsequently, DNA was eluted in water and its concentration and quality were assessed by Nanodrop 1000 microvolume 42 spectrophotometer (ThermoFisher Scientific, U.S.A.). 5 ng/µl of DNA was used to quantify the amount of mtDNA. Specifically, we measured D-loop expression, the replication start site of the mtDNA, through TaqMan Assay. RNAse-P, a sequence known to exist in two copies in human genome, was used as a reference gene. Median ΔCT per assay value was used as calibrator. TaqMAN probes used for D-loop and RNAse-P amplification are listed in **Supplementary Table S1**.

Supplemental Tables

Table S1. List of TaqMan Probes used in quantitative real-time PCR experiments.

Probes	Catalog Number
ALAS1	ThermoFisher #Hs00167441_m1
GAPDH	ThermoFisher #Hs02786621_g1
GCK	ThermoFisher #Hs01564555_m1
MT-7S (D-loop)	ThermoFisher #Hs02596861_s1
PBGD	ThermoFisher #Hs00609297_m1
PFK-L	ThermoFisher #Hs01036347_m1
PK	ThermoFisher #Hs00176075_m1
PPARGC1A	ThermoFisher #Hs00173304_m1
RNAse P	ThermoFisher #4401631

Table S2. Sequence of primers used in quantitative real-time PCR experiments.

	**Forward 5′→3′**	**Reverse 5′→3′**
DRP1	ACCCGGAGACCTCTCATTCT	TGACAACGTTGGGTGAAAAA
OPA1	TGTGAGGTCTGCCAGTCTTTA	TGTCCTTAATTGGGGTCGTTG
MFN2	CACATGGAGCGTTGTACCAG	TTGAGCACCTCCTTAGCAGAC
ACTB	GCTACAGCTTCACCACCACA	AAGGAAGGCTGGAAAAGAGC

Table S3. List of antibodies and relative dilutions used in Western blotting and ICC experiments.

Antibody	Catalog Number
COX-II (1:1000 WB)	Abcam #ab110411
CS (1:1000 WB)	Abcam #ab110216
DRP1(1:1000 WB)	Abcam #ab56788
PGC1-α (1:1000 WB)	Novus #NBP1-04676
Vinculin (1:5000 WB)	Abcam #ab73412

Supplemental Figures

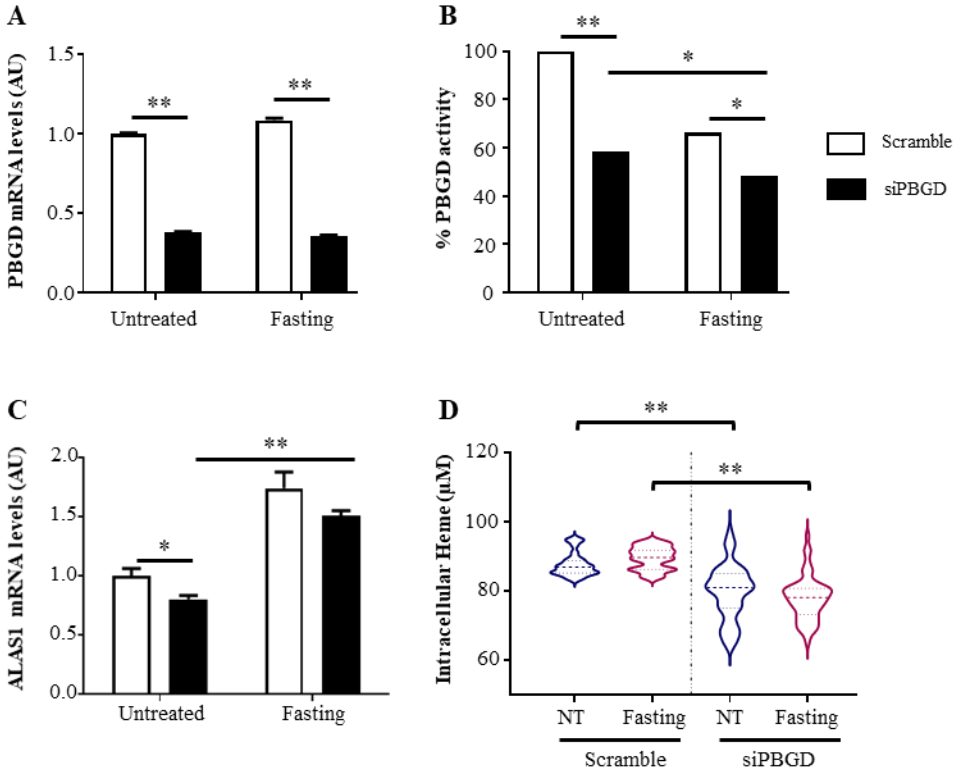

***Figure S1: PBGD downregulation reduced heme biosynthesis in HepG2 cells*.** **A**) *PBGD* mRNA expression was assessed by qRT-PCR in scramble and siPBGD cells at baseline and after fasting. **B**) PBGD kinetics was measured in both scramble and siPBGD cell lysates with or without fasting and incubated with PBG substrate for 1h. Data were normalized to mg of proteins and expressed as % of PBGD residual activity. **C**) *ALAS1* mRNA levels were assessed by qRT-PCR in scramble and siPBGD cells at baseline and after fasting. **D**) Violin plot showed intracellular heme content (μM), which was colorimetrically measured in cell lysates following the manufacturer's instruction and expressed. For gene expression, data were normalized to *ACTB* housekeeping gene and expressed as fold increase (Arbitrary Unit-AU) compared to control group. For violin plot, data were expressed as median concentration (thick dashed lines) and interquartile range (dotted lines). At least three independent experiments were conducted. Adjusted *p<0.05 and **p<0.01. PBGD: Porphobilinogen Deaminase; ALAS1: 5-aminolaevulinic acid (ALA) synthase 1; ACTB: beta-actin.

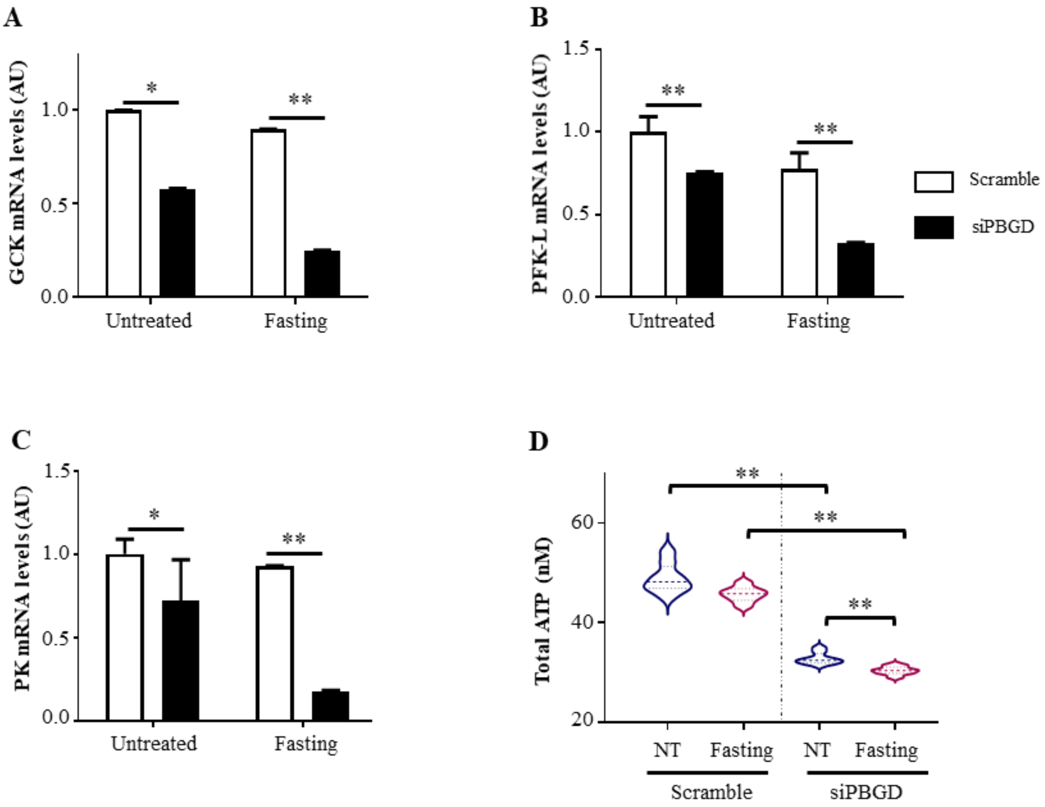

Figure S2**: *****Fasting dampened glycolysis and ATP production in siPBGD cells.*** **A-C**) *GCK*, *PFK-L* and *PK* mRNA levels were assessed by qRT-PCR in scramble and siPBGD cells at baseline and after fasting. **C**) Mitochondrial ATP concentration was quantified in cell lysates through a fluorometric assay. For gene expression, data were normalized to *ACTB* housekeeping gene and expressed as fold increase (Arbitrary Unit-AU) compared to control group. For violin plot, data were expressed as median concentration (thick dashed lines) and interquartile range (dotted lines). At least three independent experiments were conducted. Adjusted *p<0.05 and **p<0.01. GCK: Glucokinase; PFK-L: Phosphofructokinas; PK: Pyruvate Kinase; ACTB: beta-actin.

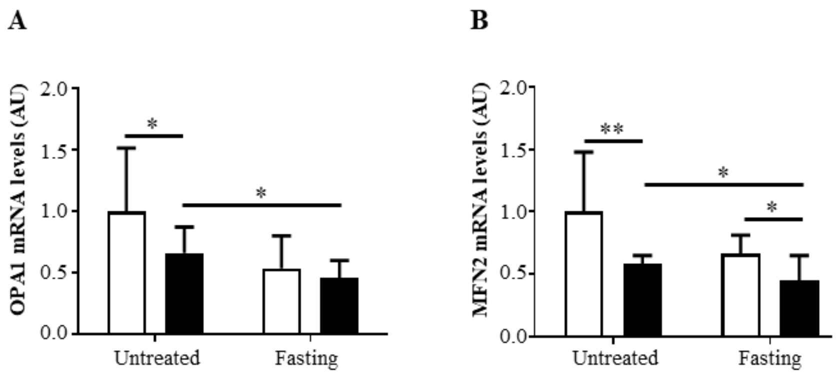

Figure S3: Fasting induced downregulation of genes involved in fusion of mitochondrial membranes. **A-B**) *OPA1* and *MFN2* mRNA levels were assessed by qRT-PCR in scramble and siPBGD cells at baseline and after fasting. Data were normalized to *ACTB* housekeeping gene and expressed as fold increase (Arbitrary Unit-AU) compared to control group. At least three independent experiments were conducted. Adjusted *p<0.05 and **p<0.01. OPA1: Optical Atrophy 1; MFN2: Mitofusin 2; ACTB: beta-actin.
